# Worms in Sheep

**Published:** 1892-01

**Authors:** Edward Stanley

**Affiliations:** Government Veterinarian, New South Wales


### WORMS IN SHEEP.*

By Edward Stanley, F. R. C. V. S., Government Veterinarian, New South Wales.

*A Paper read at the Agricultural Conference held in Sydney, July 9th, 1890.*

Mr. President and Gentlemen—Not to occupy too much of your time, I will only touch on some of the common entozoa found in our sheep, the organs they infest, the diseases they excite, concluding with preventive and remedial treatment.

As entozootic worms finally attain maturity in their host, it is seldom we find sheep entirely free from some kind or other, but they do not always cause inconvenience, and only occasionally disease and death. As a rule, parasitic worms are temporary tenants, and pass a portion of their life history independent of their hosts, the sheep. Some pass through intermediate bearers, others exist in the form of eggs or larvæ in the water, or amongst herbage, and in this form they are distributed far and wide by travelling sheep, other wandering animals, birds, and even insects, and so contaminate water-holes and herbage.

Conditions Predisposing to Worm Diseases.—It is well known that country described as cold, clayey, badly drained, wet soil, growing sour grasses, iron-bark timber, swamp oak, apple trees, and hilly districts deficient in lime, are the most favorable for parasitic life, and are the least nutritious for sheep. In such localities we find sheep die often in large numbers, from a variety of parasites. Some attack one organ, and some another, and by virtue of their multitudinous numbers they exhaust the vitality of their hosts.

Even good rich country, well drained plains, growing myall, box, salt-bush, boree, cotton-bush, etc., do not escape their invasion in very wet seasons, owing to the excessive herbage, wet camps, and moisture everywhere. Such conditions are exceedingly trying to sheep, especially to breeding ewes, lambs, and weaners, and it is these sheep that suffer most, and are thereby predisposed to diseases; they fall off in condition, and are, therefore, less able to

*"The Australasian Veterinary and Live Stock Journal."

resist an invasion of worms, which, under other and more favorable circumstances, would pass away unnoticed, and apparently without inconvenience

Symptoms of Helminthiasis, or Worm Disease.—The sheep lose spirit and activity, follow in the rear of the flock, get thin, have swollen bellies, the wool has a dead, lustreless appearance, rises on the back, and opens as the sheep walk, gets dry and harsh, being deficient in yoke ; the skin has lost the healthy, moist, greasy appearance and rosy pink color ; it gets deadly pale, clammy, and dry ; the eyes look abnormally bright and watery, the veins are nearly empty of blood ; anæmia becomes general, œdematous effusions follow, attended by a rapid loss of flesh and strength ; diarrhœa and dysentery set in, with palpitating heart and giddy head, so that when driven they rapidly become exhausted, fall, and die.

Lung Disease, caused by the *Strongylus filaria bronchialis*, a hair-like worm found inside the bronchial tubes. In addition to the above symptoms, we notice that they irritate the mucous membrane, block up small tubes, excite inflammation of the lung tissue, cause panting breathing, a hacking cough, increased mucous secretion, which is expectorated often with worms entangled, and so disseminates the ova, and spreads the disease, death being due to hepatization of lung tissue and asphyxia.

Liver Disease is caused by the *Fasciola hepatica*, in its sporadic stage called cercareæ, entering the stomach, developing larvæ flukes, these traverse up the bile ducts and invade the liver, and remain there to complete their final stage of development ; the symptoms they produce entirely depend on their numbers ; a few increase the activity of the liver, and hasten fattening, but many excite acute inflammation and rapid death ; in the chronic form of the disease they dilate the bile ducts and destroy the liver tissue till it appears rotten—this is seen by a dirty, yellow color of the skin and eyes, like jaundice ; it is accompanied by indigestion, general wasting, and dropsical swellings beneath the skin, crepitating under manipulation on the loins, and the swelling is known as "bottle" under the jaws. The ova of the flukes can be found on examining the excrement. *Post-mortem* examination shows the absence of fat, pale flesh, watery blood, serous infiltration into the connective tissues ; the liver may be studded with ecchymosed spots of blood, caused by myriads of minute flukes, but more often the bile ducts are distended like yellow pipes. On opening, these are found to contain the fluks, which are somewhat like a tea-leaf, being ovate, flat, and brownish in color.

STOMACH DISEASE in sheep is most frequently owing to the *Strongylus contortus*—a fine, hair-like worm, red in color, a true blood-sucker being attached inside the fourth stomach ; when in very large numbers they interfere with the process of digestion ; they excite chronic gastritis, as they bite like leeches, perforating the highly sensitive mucous membrane, and cause more or less hœmorrhage, indigestion, flatulency, anæmia, and death.

INTESTINAL DISEASE is principally caused by the tape worm, the *Tænia expanasa*, a very long, flat, white worm. It is made of many joints or segments, forming a colony ; each segment is sexually mature, and as they are passed in the excrement look like small white maggots ; they are the proglottides, or free form, and full of eggs.

Sometimes tape worms are sufficiently numerous in a single lamb to fill a quart pot, they do not injure the intestines, but live on the nutrient juices, cause indigestion, diarrhœa, and fatal anæmia.

Several other worms are found in sheep—the *Cœnurus tenuicollis*, a bladder worm in the abdominal cavity, and in the various organs, are sometimes very numerous, but rarely do much harm ; it is progenitor to the *Tænia marginata* of the dog.

The *Cœnurus cerebralis* is the bladder worm found in the brain, causing cranky or giddy sheep.

The *Trichocephalus affines*, the whip.shaped worm, found with its head buried in the mucous membrane of the large intestines, is also usually harmless.

The *Amphistoma conicum*, found in the first stomach, and in the sinuses of the head, is like a short, red leech, and not often fatal. *Oxyurides*—small, white worms in the colon—are also unimportant. More interesting is the *Cysticerci ovis*, the flesh worm of mutton that developes into the human *Tænia tenella*.

There are several other smaller varieties always found on searching carefully. It is the rule to find sheep suffering from Helminthiasis to be acting host to several varieties of parasites at the same time. It must always be remembered that it is the numbers of parasites that kill—some prey on the blood, some on mucus, some on bile, some on the nutrient juices, so that the poor victim in a bad season cannot nourish his own body, much less all the tenants that are in possession, therefore he wastes away and dies.

Fortunately, such evil consequences are of rare occurrence. It is contrary to the laws of nature for the host to succumb to the temporary parasite.

The Life History of parasites is of importance as indicating methods of evading their attacks, and as some of these have been ascertained, I will mention them briefly.

The liver fluke, *Fasciola hepatica*, is hermaphrodite, and finally develops in a backboned animal, and multiplies by producing eggs. One parasite contains several thousands of eggs; they are just visible as brown granules to the naked eye. After leaving the sheep each egg gives rise to the first generation, an animal, named an embryo, which is never like its parent; it enters a snail or slug, and there grows and multiplies, not by producing eggs, but by giving rise to germs within itself, which may pass through three or even four generations in the snail. These are known as Sporocysts. They finally develop into Cercaria, very minute organisms that are distributed by the snails on the herbage and in water-holes; in this form they enter the sheep's stomach and pass into the liver ducts, there becoming flukes.

The lung worm, *Strongylus filaria bronchialis*, has been raised from eggs and bred in moist earth, without undergoing any change of form; but it is found to breed and increase much more rapidly within the lungs of a living animal.

The stomach, red worm, *Strongylus contortus*, attains sexual maturity, and no doubt at certain seasons increases by breeding in the stomach, as myriads of little ones can be found free in the stomach alongside their parents; the eggs hatch in a few hours, and the little worms are sexually matured in two or three weeks. Parasitic worms can live independent of their backboned host in moist places, but they are comparatively inactive, and many perish during the extremes of hot and cold weather.

The tape-worm, *Tænia expansa*, is the common variety, often found very numerous and several yards long in lambs. When it attains maturity, and is propagated by casting off sections from its own body—these resemble small white maggots and are named proglottides; they are the free form of the parasite and hermaphrodite, being described as egg-bags, as they contain numbers of ova, so that it is easy to understand their distribution all over roads and pastures wherever sheep may travel. The eggs enter intermediate bearers, either insects or carnivorous animals; in them they develop into cystic or bladder worms, called hydatids. The head (some hydatids have many) named a scolex, is taken up by the sheep with food or water into the stomach, finding its natural home and food in the intestines, there to develop into the tape-worm.

Remedial Measures.—I think, gentlemen, you will agree with my opinion that prevention is better than cure, because any medicinal treatment can only give partially satisfactory results, as re-infection is certain, so long as the sheep are young and susceptible and the season unfavorable to them, while especially favorable to the preservation and development of the parasites.

In wet seasons lambs should be moved on to well-drained paddocks, and not be thick on the ground. Drainage of infected land is very important. Having selected the soundest land available, keep the ewes in good condition ; watch the lambs carefully for the first indications of disease ; they are often infested when only three or four weeks old, and even earlier. It is good practice to remove and separate healthy from diseased, as the latter contaminate the ground. They should be moved to sound situations, where the ova of the parasites have the least chance of development, such as salt bush, and saline licks should be given.

Salt is frequently given in rocky lumps left about the paddocks for sheep to lick. This is a very tedious process ; it causes waste of saliva, which is a fluid necessary to digestion, and the time lost in trying to get sufficient salt is required by sick sheep for eating, ruminating, and resting, therefore, given in this form, it often fails in arresting the development of worms. The kind known as Liverpool salt is in small crystals. This is the most suitable form. It should be spread out in pigeon-holed troughs, made to prevent any waste, or into long troughs of sheet-iron, bark, or hollow log. Care is necessary when first using it to prevent the sheep crowding and smothering in their eagerness to reach the salt. The supply should be renewed two or three times a week, so that they can get a little every day to eat.

The advantages of giving salt in this form are that it kills parasitic larvæ, and perhaps also eggs, and by being mixed with the food it promotes digestion by its acid reaction in the stomach. Sulphate of iron ground to a fine powder and thoroughly mixed with the salt in about the proportion of from half to one hundred weight of iron to a ton of salt, forms the best prophylactic remedy that is suitable for general use. Sulphate of iron destroys larval worms ; its astringent action stops the bleeding in the perforated mucous membrane caused by the bites of worms ; it is an element required in the formation of new blood ; in fact, this tonic action makes it of special value for worm-infested sheep ; but too much iron, or its prolonged use, will cause constipation, discolor the wool, and make it coarse. It will be most advantageously given in spring and autumn.

The treatment for lung worms is not very satisfactory, being very difficult in practice. Nevertheless, fumigation has many advocates. It is done in an enclosed, almost air-tight building by burning sulphur, and compelling the sheep to inhale the fumes. I have found it necessary to repeat the process at intervals of a week or ten days to obtain good results, about the same time giving spirits of turpentine as a drench. Chlorine and carbolic gases are sometimes used.

Intra-tracheal injection of carbolic acid, opium, and turpentine has some advocates, but it is too difficult an operation for extensive or general use. It may be valuable when properly performed in a stud flock.

The treatment for fluke is most unsatisfactory. Sheep have been kept alive on flukey country by the use of salt and sulphate of iron *eaten daily*, so destroying the young in their larval stage.

Marketable flukey sheep are best sold for slaughter while in fair condition. Many flukey sheep survive on being moved to good country, especially where salt-bush is part of the feed, but no good can be expected from drenching with any medicinal remedies, as they can only reach the liver after passing through the system with the circulation of the blood. Lime also as a lick is very beneficial in country where it is deficient, but it must be given alone, and not mixed with salt, as they are chemically incompatible.

Various drugs are used for expelling worms from the different domestic animals. In treating sheep, economy with safety is absolutely essential for success. I advocate spirits of turpentine as a vermifuge. Spirits of turpentine is widely known as the most suitable vermifuge for sheep ; it penetrates every part of the body, is exhaled from the lungs, excreted from the skin and kidneys, and promotes the action of the bowels : it is powerfully antiseptic in the blood, and is healing to the injured stomach ; it does not kill some worms outright, but it makes their residence disagreeable and the animal juices intolerably nasty; so the worms sicken, many never to recover, and in that state leave the system. It can be given in large doses by inexperienced hands without doing serious mischief. The proper way to use it is combined with some fluid with which it will blend, *i. e.*, mix thoroughly well, and at the same time be thin and flow freely. Cow's milk is the best, next equal parts of raw linseed or castor oil and thin gruel—this makes it more palatable. The dose of turpentine is from a quarter of a fluid ounce to an ounce, with twice or three times as much of the blending food in each dose, according to the age, size, and strength of the lamb. I advise this medicine to be repeated in seven days, and again fourteen days later on. Some sheep may even require a further repetition of the remedy; the most sickly should be culled, and isolated for more careful treatment in the shape of additional nutritious feed, plenty of salt, &c.

There are 20 fluid ounces in one pint ; one fluid ounce is about equal to two tablespoonfuls.

ARSENIC AS A REMEDY.—Some persons advocate arsenic ; it will kill worms and sheep also. Given to sheep in grain doses, and in solution, it is said to act satisfactorily (personally, I have no experience of its use as an anthelmintic), but from the state of a sheep's stomach infested with worms, I should fear gastritis and constitutional disturbance; therefore the rumor that arsenic injures the wool requires investigation, and would imply that considerable care is necessary in its use.

CARE IN DRENCHING.—In conclusion I may point out that sheep, being timid and rapid-breathing animals, require more than ordicare in drenching, otherwise they are easily choked, even with milk or gruel. It must be remembered a sheep drinks (that is, swallows) with his head held low. To place him in a natural position, he should sit upright on the ground, his back supported between the knees of the man holding him ; the mouth should only be elevated just enough to allow the drench to flow from the bottle to the throat. The neck of the bottle should be long and narrow, to enter the mouth easily, and the opening small, so as to flow slowly. A taste of the drench should be given first, and as soon as it is swallowed the remainder may follow. It is a mistake to fill the mouth too full or to try and arrest a coughing fit; it is better to let the sheep go and waste the dose than to risk killing him. Sheep bear yarding and handling best with empty stomachs, and medicine is more effective given then.

If it is necessary to give one dose, it is advisable to repeat it at intervals, as there may be thousands of worms to remove, and reinfection is constantly going on, at least during the active breeding seasons of the worms, and some young sheep are sure to suffer from the attacks to a greater extent than others ; they should be marked and treated separately.

From this paper it will be seen that treatment for worms is not entirely satisfactorily, therefore prevention is better than cure. As there are several sheep-owners present, it will be interesting to hear their experiences of treatment, and a general discussion will diffuse information.

